# Personality Profile in Focal Hand Dystonia: A Cross-Sectional Study

**DOI:** 10.3390/ijerph18157863

**Published:** 2021-07-25

**Authors:** Marta Pérez-de-Heredia-Torres, Elisabet Huertas-Hoyas, Nuria Trugeda-Pedrajo, Sergio Serrada-Tejeda, Alfonso Gómez-Gil-Díaz-Río, Juan C. Martínez-Castrillo

**Affiliations:** 1 Physical Therapy, Occupational Therapy, Rehabilitation and Physical Medicine Department, Rey Juan Carlos University, Av. Atenas s/n, 28922 Madrid, Spain; marta.perezdeheredia@urjc.es (M.P.-d.-H.-T.); nuria.trugeda@urjc.es (N.T.-P.); sergio.tejeda@urjc.es (S.S.-T.); 2 Community Occupational Therapy Team, Essex Partnership Trus, National Health Service, Grays Hall, Cm12ws, Chelmsford RM17 5TT, UK; a.gomez-gildiaz-rio@nhs.net; 3 Movement Disorders Unit, Department of Neurology, Universitario Ramón y Cajal Hospital, 28034 Madrid, Spain; jmcastrillo@salud.madrid.org

**Keywords:** activities of daily living, dystonia, emotional stability, movement disorder, participation, personality traits

## Abstract

It has been suggested that focal hand dystonia (FHD) should be viewed as a neuropsychiatric disorder rather than as a pure movement disorder. We aimed to compare the personality factors that are common to people with FHD and evaluate how personality factors could affect the functionality of the upper limbs and community participation. We conducted a cross-sectional case–control study in which 12 people with FHD were matched with 12 age and gender matched healthy control participants. The Big Five Questionnaire; the Quick Disabilities, Arm, Shoulder, and Hand questionnaire; and the Jebsen–Taylor Test of Hand Function were used as assessment measures. Control of emotions was the only variable for which a significant difference was found, with participants with FHD displaying lesser control. Correlations were not observed between different personality profiles, the functionality of the upper limbs and the perceived participation of people with FHD in activities of daily living. People with FHD may present with low emotional stability, but this does not have a negative impact on the functionality of the upper limbs and activities of daily living. These findings have clinical implications to be considered for interventions, as they suggest that personality aspects, such as extraversion, may not predict for better functionality and perceived participation in activities of daily living.

## 1. Introduction

Dystonia is a functional movement disorder (FMD) characterized by the presence of sustained or intermittent agonist and antagonist muscles contractions and leading to repetitive movements, abnormal postures, or both [1]. When dystonia affects an isolated single body region, it is known as focal dystonia.

Most people with focal dystonia perceive a high level of stigma associated with the condition, which can affect their social and private life as well as their work environment. This perceived stigma should, therefore, be considered a relevant parameter in the clinical management of affected patients [2]. Focal dystonia is associated with numerous symptoms and/or disorders, such as depression, anxiety, specific phobia, perfectionism, and neuroticism. Some authors [3] point to the possibility that motor and psychiatric symptoms may be an underlying isolated focal dystonia, which may reflect different mechanisms of the disease. These psychiatric comorbidities, frequently associated with anxious and depressive symptoms, are quite common and are considered independent elements of the disease [4]. They are known as non-motor symptoms of focal dystonia. The appearance of this psychiatric symptomatology may be a consequence of the disorder (FMDS) characterized and may not play a role in the etiology of the disease [5]. It has recently been seen that the presence of stressful events during childhood or adulthood are more common in patients with FMD disease [6], which may be associated with the appearance of anxiety or depressive disorders as well as the appearance of personality disorders [7].

Lencer et al. [8] found several personality traits among people with focal dystonia when compared with unaffected individuals, including greater awareness and kindness and less open-mindedness. The fact that different personality profiles have been identified in different types of focal dystonia has raised new questions. Some authors [7,9] point to the possibility of certain personality traits contributing to the decision of choosing certain professions and lifestyles—that is, that the dystonia is somehow the consequence of their main occupation.

Personality traits help distinguish individual differences in terms of characteristic qualities, thoughts, or behaviors [10]. One of the most important models for describing personality is the *Five-Factor Model*, which assesses specific personality traits. The interpretation of the same is based on five factors: openness, conscientiousness, extraversion, agreeableness, and emotional stability. These dimensions are accurate, valid, and reliable and have been used extensively [11].

Steinlechner et al. [9] suggest a specific personality profile for musician’s dystonia, which may match with other types of focal dystonia; Lencer et al. [8] suggest that focal dystonia should be approached as a neuropsychiatric disorder instead of a pure movement disorder. If this is the case, the fact that people with FHD present a specific personality tendency can lead to the question: how does this affect the functionality of the upper limbs, along with activities of daily living and community involvement? So far, no one has published data about this issue. Therefore, this study sought to compare the different personality factors which are common to people with FHD to those of people without dystonia, as well as to evaluate possible correlations between personality factors, the functionality of the upper limbs, and participation of people with FHD in activities of daily living.

## 2. Materials and Methods

### 2.1. Study Design

A preliminary observational cross-sectional case–control design was used. The study was approved by the Ethics Committee of the Rey Juan Carlos University and by Ramón y Cajal Hospital (n° 160320162916) and was conducted in accordance with the Helsinki Declaration of Human Rights.

### 2.2. Sample

Consecutive, non-probabilistic sampling was used for the group of people with FHD, recruited from the Unit of Movement Disorders of the Neurology Service of the Ramón y Cajal Hospital. Convenience sampling and age-sex matched pairs were used to recruit the control group. All participants were recruited voluntarily and gave informed consent. All patients were aware that they could abandon the study at any time. In the case of patients with FHD, their respective neurologist informed prospective participants about this study and provided informed consent. For the recruitment of healthy participants, the study was advertised via signs placed at both the Faculty of Health Sciences and the university clinic.

The selection criteria for participants with FHD were as follows: patients should be presenting a FHD diagnosed with isolated dystonia of the upper limbs of a focal hand type, task-specific, or rest tremor, according to the consensus criteria of clinical diagnosis from the Movement Disorder Society [12], with the involvement of the hand; be right-handed; be receiving treatment with periodic infiltrations of botulinum toxin type A, for which the last treatment was at least 4 months ago, without any other drug with effects on the central nervous system; be negative for DYT-1; sign the informed content; not be suffering from any other concomitant illnesses; be over the age of 18 years.

### 2.3. Measurement Tools

Each participant was administered the following outcome measures:

- Big Five Questionnaire (BFQ): Designed by Caprara et al. [13] in 1993 and adapted to the Spanish population by Bermúdez [14]. This test aims to measure the five principal (“Big Five”) dimensions of human personality and was one of the first personality questionnaires developed in Europe. The BFQ consists of 132 multiple response items (Likert scale), which measure the following dimensions: extraversion (inherent to a trusted and enthusiastic vision of multiple aspects of life, mainly of an interpersonal type), which dynamism and dominance belong to; agreeableness (altruist concern and emotional support for others), which cooperation and friendliness belong to; Conscientiousness (a perseverant type of behavior, scrupulous and responsible), which scrupulousness and perseverance belong to; emotional stability (wide spectrum trait, characterized by the ability to face the negative effects of anxiety, depression, irritability or frustration), which control of emotions and impulses belong to; and openness (in terms of openness towards new ideas, values, feelings, and interests), which cultural and experience openness belong to. It also includes a scale of distortion, which is useful for detecting possible attempts to convey a false image, good or bad, on behalf of the subject. The data are introduced into the designated software, indicating each person’s profile according to the strength of the different dimensions.
- Quick Disabilities, Arm, Shoulder and Hand (QuickDASH): This tool is based on 11 items, compared with the 30 original items, for the measurement of physical function and symptoms in people with any kind of musculoskeletal disorder in the upper limbs [15]. To calculate the score, at least 10 of the 11 questions must be completed. The assigned values for all the completed responses are summed and the mean value calculated, which provides a score from 1–5. This value is later transformed to a score out of 100, subtracting 1 and multiplying by 25. The higher the score, the greater the disability and the lower their participation in the community. The analysis of the psychometric properties is evaluated by means of Cronbach’s alpha coefficient, whose value can vary from 0 to 1; it is considered significant when it is higher than 0.70. The value obtained in this study was Cronbach’s alpha = 0.839.
- Jebsen–Taylor Test of Hand Function (JTTHF): The JTTHF assesses a wide range of manual functions simulating certain activities of daily living. It consists of seven subtests (writing, simulating turning pages, lifting small common objects, simulated feeding, stacking checkers, lifting large light objects, lifting large heavy objects), all of which are timed. Each activity is performed alternating the dominant and non-dominant hand [16]. The analysis of the psychometric properties is evaluated using Cronbach’s alpha coefficient, whose value can vary from 0 to 1; it is considered significant when it is higher than 0.70. The value obtained in this study was Cronbach’s alpha = 0.613.

Regarding data collection, we included the severity of dystonia using the Unified Dystonia Rating Scale (UDRS) [17], sociodemographic data, and retrospective clinical profiles for the more affected side (musculoskeletal and psychoemotional problems and anxiety-depressive symptoms) using semi-structured interviews in order to obtain global patient status information.

### 2.4. Data Analysis

Firstly, the analysis of the normality of the sample was performed using the Shapiro–Wilk test. Thereafter, the descriptive statistics and homogeneity of variance were calculated for both samples using Levene’s test. Secondly, descriptive data were obtained to evaluate the frequency of the categorical variables, together with the mean and the standard deviation of the remaining continuous variables. After confirming the homogeneity of the sample, parametric tests were performed to examine the differences between independent samples and their correlations. Subsequently, the possible differences between the independent variables of both comparison groups were evaluated using a Student’s *t*-test. Differences in proportions were calculated using Pearson’s Chi-squared test. The *p* value was *p* < 0.05.

The Multivariate analysis of variance (MANOVA) was used to compare the BFQ scale between the groups, followed by continuation Analysis of variance (ANOVA)when the MANOVA was significant.

For the size of the effect, we considered Cramer’s V: 0.10, small; 0.30, medium; 0.50, large. eta squared: 0.01, small; 0.06, medium; 0.14, large. Cohen’s d (dr): 0.20, small; 0.50, medium; 0.80, large [18]. Lastly, the possible correlations between variables were confirmed via Pearson’s correlation coefficient, along with Bonferroni’s correction for multiple comparisons to control for the error rate and the probability of making a type I error. The application of the Bonferroni correction allowed us to base our decisions on a level of significance of 0.05/(15 × 5) = 0.0006, considering correlations with a value less than 0.000 (*p* < 0.0006) to be significant.

The analysis of variables was performed using the IBM SPSS Statistics statistical program for Windows, version 26 (Copyright© 2013 IBM SPSS Corp.), adding a syntax for the estimation of the effect size. The level of significance was *p* < 0.05.

## 3. Results

Table 1 displays the descriptive data of both groups—those with FHD (n = 12) and the group without dystonia (n = 12)—as well as the various tests administered. Additionally, it shows the differences between both groups, with the FHD group having more musculoskeletal and psychoemotional problems in a global condition than the no dystonia group (see Supplementary Table S1 for the manual dexterity of the sample).

For the five dimensions of the BFQ, the MANOVA test showed that there were no statistically significant differences between patients (Hotelling’s trace: F (5; 18) = 0.628, *p* = 0.681, eta2 = 0.019). However, MANOVA tests performed for the sub-dimensions showed significant differences between patients (Hotelling’s trace: F (10; 13) = 2711, *p* = 0.048, eta2 = 0.412). According to the continuation ANOVA, the differences were shown in the emotion control dimension, where the score of the patients without dystonia was significantly higher than that of patients with dystonia. In the other dimensions, no statistically significant differences were observed.

Table 2 shows the frequencies of the five main personality dimensions for the different groups (with and without FHD), as well as the comparative analysis, revealing a lack of significant differences between the two groups.

A Chi-squared analysis of the differences between the variables of both study groups revealed that the only variable displaying a significant difference was that of the control of emotions, with the effect size being very high (dr = 0.93), observing lower control in the group of people with FHD (Table 3). However, if we look at the effect size instead of the *p* value, this indicates that there are other differences between low and high values in other aspects, such as cultural openness (dr = 0.26), dynamism (dr = 0.37), and emotional stability (dr = 0.66), respectively.

Regarding the possible correlations between different personality profiles, the functionality of the upper limbs, and the perception of participation in activities of daily living (via the Jebsen and QuickDash tests, respectively) of people with FHD, the results indicated that there were no correlations when considering the Bonferroni correction (see Supplementary Table S2 for correlations).

## 4. Discussion

The main aim of this study was to describe the different personality factors of people with FHD and to find out the correlation of the same with regard to daily functioning. According to the data found in our study, no significant differences were found regarding personality factors in people with FHD and those without FHD, except for the control of emotions. Furthermore, although the difference was non-significant when comparing the frequencies of the personality factors among both groups, a difference was found regarding emotional stability, which was lower in people with FHD. This means that people without FHD may present with a greater ability to face depression, irritability, frustration, and the negative effects of anxiety when compared to people with FHD. Our results are probably limited by the low sample size used. However, according to our results, the global status is already different between both groups because there is a higher frequency of anxiety in the dystonia group than in the no dystonia group. This is in line with the results of Caprara et al. [13], who reported that the characteristics of this personality trait enable people to face depression and anxiety. These findings are similar to those reported in a paper by Likhachev et al. [19] on personality profiles, whereby a high level of anxiety was reported in patients with dystonia, as well as increasing emotional instability and poor emotional control. Likewise, several authors [3,9,20,21] have detected increased anxiety, social phobia, perfectionism, and/or neuroticism in musicians. The reason for this remains unknown; however, it is easy to understand how suffering an illness such as dystonia can trigger feelings of anxiety and/or depression, as this affects a person’s activities of daily living, compared with people without this disorder. Specifically, in the case of musicians, the illness represents an added burden, as it negatively affects their performance in playing an instrument. Additionally, it would be interesting to analyze and compare with other kinds of isolated dystonia such as cervical dystonia, laryngeal dystonia, or blepharospasm because they could have similar risk and patterns, as Berman et al. indicated [22]. Whether they have similar patterns or not, all these types of isolated dystonia could have similar psychoemotional conditions or even personality factors. A recent interesting article by Eggink et al. [23] shows how non-motor symptoms, such as anxiety or depression symptoms, already appear in children and young adults with dystonia, suggesting that non-motor symptoms play an important role in the phenotypic characterization of dystonia.

Regarding the relationships analyzed between the functionality of the upper limbs, perceived participation in activities of daily living, and personality profiles, it appears that there is no relationship between them, so this emotional stability trait may not affect the functionality of the upper limbs or activities of daily living. Perhaps a possible reason for this is the sample size, but according to our results personality factors such as extraversion, control of emotions, or kindness may not affect the functionality of the upper limbs and, therefore, participation of daily life activities. This belief is not in consistent with the findings of Jabeen et al. [24], who considered the study of personality traits to potentially be able to improve our ability to make a more accurate prognosis of recovery and help expose the etiology of mental illnesses. In their study, the personality traits of different students with and without physical disability (no FHD) were analyzed. Thus, according to Jabeen et al. [24], people with disability display significant differences in their personality traits compared to people without disability, as they are more susceptible to suffering from anxiety and substance abuse and have a greater risk of depression. Furthermore, as indicated, physical disability has a profound effect on quality of life, specifically on a person’s social relations and emotional wellbeing. This could lead us to question whether it is a type of personality which leads to a person being more vulnerable to certain pathologies, such as dystonia, as already postulated by Lencer et al. [8] or vice versa—for example, whether suffering a disorder such as dystonia can lead to the development of a certain type of behavior/personality. This last hypothesis appears quite weak, however, because, as indicated by the authors [10,25], personality is based on character, which describes and predicts an individual’s behavior in different situations, whereas psychological traits are stable and structured [24]. As noted by Berardelli et al. [26], although psychiatric disorders are frequent in hyperkinetic movement disorders, few controlled studies have evaluated the treatment of psychiatric disorders in these disorders.

As indicated by a recent review [27], we have progressed in our understanding of the etiology, risk factors, and pathophysiology of focal task-specific dystonia, but improved therapeutic options are needed. Thus, it is important to consider these results, as they may be useful for guiding interventions, not so much from the perspective of motor functioning in activities of coordination and fine dexterity, but rather by focusing on personality factors such as emotional stability. Although, according to our results, the lack of functionality during activities of daily living may not be affected by emotional instability, activities that require fine or complex dexterity or even self-perception could cause anxiety, as indicated by Cylan et al. [28] and Martínez-Piédrola et al. [29]. As indicated by Mahaffey et al. [30] in their study, certain clinical traits (perfectionism, sensitivity to anxiety, among others) increase the risk and chronicity of emotional disorders. Interestingly, these traits coincide with another type of dystonia, as mentioned previously [3,9,19,20,21]. Furthermore, these authors suggest that the study of the clinical traits underlying emotional disorders is both relevant and valuable for predicting psychopathology. Thus, it is very important to consider this regarding targeted interventions with FHD.

This study presents several limitations. The sample size was small; therefore, the results should be interpreted with caution. Nonetheless, considering the specificity of this pathology there were many difficulties for accessing and recruiting a large sample. Additionally, most of the participants were male, causing the sample to be unbalanced. It would be very interesting to have a control group with functional movement disorders in order to discuss the differences and similarities of dystonic patients. In addition, we consider that there are limitations to the results due to the use of tools that have not been validated in a population with dystonia. However, we have chosen one of the most commonly used tools for upper limb motor deficits.

## 5. Conclusions

The clinical implications of this preliminary study are that a large percentage of people with FHD present with a low emotional stability factor, but apparently this may not affect the functionality of the upper limbs in activities of daily living. These results may be due to the limited sample size used in this study. Therefore, it is advisable to increase the sample size to provide more information on the variables studied. However, it is necessary for interventions to be directed—not so much at motor aspects of the upper limbs, but also considering personality aspects such as extraversion or emotional control, as well as to see whether this treatment approach improves perceived participation in activities of daily living. Future research with a larger sample size should consider these aspects.

## Figures and Tables

**Table 1:** Characteristics of the sample, focal hand dystonia (n = 12) and no dystonia (n = 12).

	FHD	No Dystonia	*p*-Value	Effect Size
Age, mean ± SD	51.50 ± 16.80	50.08 ± 12.2	0.816 ^‡^	0.096 _a_
Duration of symptoms, mean ± SD	13 ± 14.74	-		
UDRS	2.17 ± 0.68	-		
Sex, n (%)			0.5 ^†^	0.092 _b_
Women	3 (25)	4 (33.3)		
Men	9 (75)	8 (66.7)		
Dominance, n (%)				
Right-handed	12 (100)	12 (100)		
Left-handed	0 (0)	0 (0)		
More affected side, n (%)				
Right-handed	12 (100)	0 (0)		
Left-handed	0 (0)	0 (0)		
Musculoskeletal problems, n (%)			0.014 ^†^	0.577 _b_
Neck	2 (16.7)	0 (0)		
Right shoulder/	3 (25)	0 (0)		
Lumbar spine/	1 (8.3)	1 (9.1)		
None	6 (50)	11 (90.9)		
Psychoemotional problems, n (%)			0.083 ^†^	0.469 _b_
Anxiety	4 (33.3)	1 (8.3)		
Frustration	1 (8.3)	0 (0)		
Combination	1 (8.3)	0 (0)		
None	6 (50)	11 (91.7)		

NOTE: ^‡^ Student’s *t* test. ^†^ Fisher’s Exact Test. _a_: Cohen’s d. _b_: Cramer’s V.

**Table 2:** Description of the sample by dimensions and Chi-squared comparison.

Score	Extraversion, *n* (%)		Agreeableness, *n* (%)		Conscientiousness, *n* (%)		Emotional Stability, *n* (%)		Openness, *n* (%)	
	Dystonia		Dystonia		Dystonia		Dystonia		Dystonia	
	Yes	No	Yes	No	Yes	No	Yes	No	Yes	No
Very low	3 (25)	3 (25)	3 (25)	1 (8.3)	1 (8.3)	3 (25)	3 (25)	1 (8.3)	1 (8.3)	0 (0)
Low	7 (58.3)	7 (58.3)	4 (33.3)	4 (33.3)	4 (33.3)	3 (25)	7 (58.3)	4 (33.3)	5 (41.7)	5 (41.7)
Average	2 (16.7)	1 (8.3)	3 (25)	4 (33.3)	4 (33.3)	3 (25)	1 (8.3)	5 (41.7)	4 (33.3)	5 (41.7)
High	0 (0)	1 (8.3)	0 (0)	1 (8.3)	3 (25)	3 (25)	1 (8.3)	2 (16.7)	2 (16.7)	2 (16.7)
Very high	0 (0)	0 (0)	2 (16.7)	2 (16.7)	0 (0)	0 (0)	0 (0)	0 (0)	0 (0)	0 (0)
Pearson’s chi-squared (Sig)	1.333 (0.721)		2.143 (0.710)		1.286 (0.733)		4.818 (0.186)		1.111 (0.774)	

**Table 3:** Descriptive results and ANOVA (BFQ).

	FHD	No Dystonia	Continuation ANOVA’s		
	Mean ± SD	Mean ± SD	F (1;22)	*p*-Value	eta^2^
Extraversion	39 ± 6.31	40.1 ± 7.61			
-Dynamism	40.92 ± 4.64	43 ± 6.36	0.909	0.351	0.04
-Dominance	39.17 ± 9.62	40.8 ± 8.02	0.212	0.65	0.01
Agreeableness	46.83 ± 11.5	48.2 ± 10.34			
-Cooperation	49.08 ± 12.6	48.8 ± 9.82	0.003	0.957	0
-Friendliness	45.83 ± 10.7	47.1 ± 11.16	0.089	0.768	0.004
Conscientiousness	47.42 ± 8.61	45.9 ± 11.54			
-Scrupulous	51.58 ± 9.61	51.25 ± 9.14	0.008	0.931	0
-Perseverance	42.33 ± 7.7	41.5 ± 10.7	0.048	0.829	0.002
Emotional stability	40.42 ± 8.45	46.4 ± 9.62			
-Control of emotions	38.33 ± 7.19	46.08 ± 9.24	5.252	0.032	0.193
-Control of impulses	45.33 ± 10.4	47.4 ± 8.91	0.277	0.604	0.012
Openness	46.08 ± 9.99	47.5 ± 7.15			
-Cultural openness	46.5 ± 11.8	49.1 ± 6.9	0.452	0.508	0.02
-Experience openness	46 ± 9.07	46.6 ± 10.9	0.026	0.872	0.001
Distortion	52.25 ± 8.22	49.9 ± 10.05			

NOTE: eta^2^: effect size. The homogeneity of the variance-covariance matrices between the groups was used with the Box M contrast, previously checking the univariate homogeneity of the variance between groups using Levene’s test. The results evidenced compliance with both assumptions.

## Data Availability

Data available on request due to privacy restrictions. The data presented in this study are available on request from the corresponding author.

## Supplementary Materials

The following are available online at https://www.mdpi.com/article/10.3390/ijerph18157863/s1: Table S1: Descriptive results of manual dexterity measures; Focal Hand Dystonia (n = 12) and No Dystonia (n = 12), Table S2: correlations between different personality profiles, functionality of the upper limb and the perception of participation in activities of daily living.
