# Supplementary material for: Personality Profile in Focal Hand Dystonia: A Cross-Sectional Study
Source: Int J Environ Res Public Health. 2021 Jul 25;18(15):7863. doi: 10.3390/ijerph18157863 (PMC8345782; doi:10.3390/ijerph18157863)
Supplement: Supplementary file 1 [file ijerph-18-07863-s001.zip › ijerph-1298228-supplementary.pdf]

Supplementary Materials

Table S1. Descriptive results of manual dexterity measures; Focal Hand Dystonia (n = 12) and No Dystonia (n = 12).

QuickDASH	Distonía (n = 12)	No distonía (n = 12)
	**24.03 ± 23**	**3.18 ± 4.36**
**Jebsen Taylor Test of Hand Function**		
JTTHF_1_D, *mediana (q1–q3)*	19.93 (13.04–44.88)	10.08 (9.50–12.83)
JTTHF_1_ND, *media (DE))*	38.31 (16.33)	30.08 (12.50)
JTTHF_2_D, *mediana (q1–q3)*	5.04 (4.31–6.97)	3.67 (3.39–4.09)
JTTHF_2_ND, *mediana (q1–q3)*	5 (3.85–6.17)	4.07 (3.63–4.65)
JTTHF_3_D, *media (DE)*	6,63 (1.24)	6.67 (2.32)
JTTHF_3_ND, *mediana (q1–q3)*	6.91 (6.25–7.79)	5.99 (5.83–6.58)
JTTHF_4_D, *mediana (q1–q3)*	7,28 (5,79–15,05)	7 (5.12–9.78)
JTTHF_4_ND, *media (DE)*	9.96 (1.69)	10.22 (3.98)
JTTHF_5_D, *mediana (q1–q3)*	2.65 (1.84–5.32)	1.94 (1.55–2.30)
JTTHF_5_ND, *mediana (q1–q3)*	2.92 (2.42–4.53)	2.19 (1.74–3.07)
JTTHF_6_D, *mediana (q1–q3)*	3.88 (3.41–5.10)	3.26 (2.67–3.57)
JTTHF_6_ND, *mediana (q1–q3)*	4.07 (3.54–4.46)	3.50 (3.22–3.91)
JTTHF_7_D, *mediana (q1–q3)*	3.44 (3.08–4.61)	3.38 (2.71–3.71)
JTTHF_7_ND, *mediana (q1–q3)*	3.65 (3.30–4.22)	3.52 (2.90–4.04)

NOTE: JTTHF_1_D: Jebsen-Taylor Hand Function Test, dominant side writing; JTTHF_1_ND: Jebsen-Taylor Hand Function Test non-dominant side writing; JTTHF_2_D: Jebsen-Taylor Hand Function Test, pass leaves dominant side; JTTHF_2_ND: Jebsen-Taylor Hand Function Test, pass leaves non-dominant side; JTTHF_3_D: Jebsen-Taylor Hand Function Test, small objects dominant side; JTTHF_3_ND: Jebsen-Taylor Hand Function Test, small objects non-dominant side; JTTHF_4_D: Jebsen-Taylor Hand Function Test, similar power supply dominant side; JTTHF_4_ND: Jebsen-Taylor Hand Function Test similar power supply non-dominant side; JTTHF_5_D: Jebsen-Taylor Hand Function Test, stacking chips on dominant side; JTTHF_5_ND: Jebsen-Taylor Hand Function Test, stacking chips on non-dominant side; JTTHF_6_D: Jebsen-Taylor Hand Function Test, move light objects dominant side; JTTHF_6_ND: Jebsen-Taylor Hand Function Test, move light objects non-dominant side; JTTHF_7_D: Jebsen-Taylor Hand Function Test, move heavy objects dominant side; JTTHF_7_ND: Jebsen-Taylor Hand Function Test, move heavy objects non−dominant side.

Table S2. correlations between different personality profiles, functionality of the upper limb and the perception of participation in activities of daily living.

	BFQ Extraversion	BFQ Agreeableness	BFQ Conscientiousness	BFQ_ Emotional Stability	BFQ_ Openness	QuickDASH
**JTHFT_1_ND**	r = −.122<br>p = 0,544	r= −0.189<br>p = 0.344	r = 0.058<br>p = 0.774	r = −0.418<br>p = 0.030	r = −0.319<br>p = 0.104	r = 0.598<br>p = 0.001
**JTHFT_1_D**	r = −0,191<br>p = 0,340	r= −0.241<br>p = 0.226	r = −0.016<br>p = 0.938	r = −,374<br>p = 0.054	r = −0.424<br>p = 0.028	r = 0.557<br>p = 0.003
**JTHFT_2_ND**	r = −0.200<br>p = 0.318	r= −0.477<br>p = 0.012	r = −0,300<br>p = 0.129	r = −0.284<br>p = 0.151	r = −0.315<br>p = 0.109	r = 0.258<br>p = 0.194
**JTHFT_2_D**	r = −0.161<br>p = 0.421	r= −0.405<br>p = 0.036	r = −0.185<br>p = 0.355	r = −0.261<br>p = 0.189	r = −0.211<br>p = 0.292	r = 0.298<br>p = 0.132
**JTHFT_3_ND**	r = −0.024<br>p = 0.904	r= −0.447<br>p = 0.019	r = −0.017<br>p = 0.935	r = −0.413<br>p = 0.032	r = −0.075<br>p = 0.708	r = 0.391<br>p = 0.044
**JTHFT_3_D**	r = 0.165<br>p = 0.409	r= −0.230<br>p = 0.249	r = 0.177<br>p = 0.377	r = −0.046<br>p = 0.821	r = 0.324<br>p = 0.099	r = 0.073<br>p = 0.716
**JTHFT_4_ND**	r = −0.363<br>p = 0.063	r = −0.243<br>p = 0.222	r = −0.315<br>p = 0.109	r = 0.087<br>p = 0.666	r = −0.243<br>p = 0.222	r= −0.034<br>p = 0.867
**JTHFT_4_D**	r = −0.141<br>p = 0.483	r = −0.229<br>p = 0.252	r = −0.373<br>p = 0.055	r = −0.038<br>p = 0.851	r = −0.185<br>p = 0.356	r = −0.061<br>p = 0.761
**JTHFT_5_ND**	r = −0.018<br>p = 0.928	r = −0.201<br>p = 0.315	r = 0.155<br>p = 0.439	r = −0.248<br>p = 0.212	r = −0.023<br>p = 0.909	r = 0.201<br>p = 0.316
**JTHFT_5_D**	r = − 0.217<br>p = 0.278	r = −0.215<br>p = 0.282	r = 0.207<br>p = 0.300	r = −0.118<br>p = 0.557	r = 0.129<br>p = 0.521	r = 0.377<br>p = 0.052
**JTHFT_6_ND**	r = −0.310<br>p = 0.115	r = −0.076<br>p = 0.705	r= −0.224<br>p = 0.261	r = −0.386<br>p = 0.047	r = 0.010<br>p = 0.962	r = 0.305<br>p = 0.122
**JTHFT_6_D**	r = −0.296<br>p = 0.134	r = −0.268<br>p = 0.176	r= −0.029<br>p = 0.886	r = −0.279<br>p = 0.159	r = −0.045<br>p = 0.822	r = 0.446<br>p = 0.020
**JTHFT_7_ND**	r = −0.302<br>p = 0.126	r = −0.180<br>p = 0.369	r = 0.022<br>p = 0.912	r = −0.246<br>p = 0.216	r = 0.106<br>p = 0.598	r = 0.215<br>p = 0.282
**JTHFT_7_D**	r = −0.308<br>p = 0.118	r = −0.297<br>p = 0.133	r = −0.010<br>p = 0.959	r = −0.331<br>p = 0.091	r = 0.025<br>p = 0.903	r = 0.263<br>p = 0.184

NOTE: JTTHF_1_D: Jebsen-Taylor Hand Function Test, dominant side writing; JTTHF_1_ND: Jebsen-Taylor Hand Function Test non-dominant side writing; JTTHF_2_D: Jebsen-Taylor Hand Function Test, pass leaves dominant side; JTTHF_2_ND: Jebsen-Taylor Hand Function Test, pass leaves non-dominant side; JTTHF_3_D: Jebsen-Taylor Hand Function Test, small objects dominant side; JTTHF_3_ND: Jebsen-Taylor Hand Function Test, small objects non-dominant side; JTTHF_4_D: Jebsen-Taylor Hand Function Test, similar power supply dominant side; JTTHF_4_ND: Jebsen-Taylor Hand Function Test similar power supply non-dominant side; JTTHF_5_D: Jebsen-Taylor Hand Function Test, stacking chips on dominant side; JTTHF_5_ND: Jebsen-Taylor Hand Function Test, stacking chips on non-dominant side; JTTHF_6_D: Jebsen-Taylor Hand Function Test, move light objects dominant side; JTTHF_6_ND: Jebsen-Taylor Hand Function Test, move light objects non-dominant side; JTTHF_7_D: Jebsen-Taylor Hand Function Test, move heavy objects dominant side; JTTHF_7_ND: Jebsen-Taylor Hand Function Test, move heavy objects non-dominant side.
